# Automatic Extraction of Water and Shadow from SAR Images Based on a Multi-Resolution Dense Encoder and Decoder Network

**DOI:** 10.3390/s19163576

**Published:** 2019-08-16

**Authors:** Peng Zhang, Lifu Chen, Zhenhong Li, Jin Xing, Xuemin Xing, Zhihui Yuan

**Affiliations:** 1 School of Electrical and Information Engineering, Changsha University of Science & Technology, Changsha 410114, China; 2 Laboratory of Radar Remote Sensing Applications, Changsha University of Science & Technology, Changsha 410014, China; 3 School of Engineering, Newcastle University, Newcastle upon Tyne NE1 7RU, UK; 4 College of Geological Engineering and Geomatics, Chang’an University, Xi’an 710054, China; 5 School of Traffic & Transportation Engineering, Changsha University of Science & Technology, Changsha 410114, China

**Keywords:** water extraction, shadow extraction, deep learning, synthetic aperture radar (SAR), classification, convolutional neural network (CNN), global convolutional network (GCN), dense convolutional network (DenseNet), CONVOLUTION LONG SHORT-TERM MEMORY (ConvLSTM)

## Abstract

The water and shadow areas in SAR images contain rich information for various applications, which cannot be extracted automatically and precisely at present. To handle this problem, a new framework called Multi-Resolution Dense Encoder and Decoder (MRDED) network is proposed, which integrates Convolutional Neural Network (CNN), Residual Network (ResNet), Dense Convolutional Network (DenseNet), Global Convolutional Network (GCN), and Convolutional Long Short-Term Memory (ConvLSTM). MRDED contains three parts: the Gray Level Gradient Co-occurrence Matrix (GLGCM), the Encoder network, and the Decoder network. GLGCM is used to extract low-level features, which are further processed by the Encoder. The Encoder network employs ResNet to extract features at different resolutions. There are two components of the Decoder network, namely, the Multi-level Features Extraction and Fusion (MFEF) and Score maps Fusion (SF). We implement two versions of MFEF, named MFEF1 and MFEF2, which generate separate score maps. The difference between them lies in that the Chained Residual Pooling (CRP) module is utilized in MFEF2, while ConvLSTM is adopted in MFEF1 to form the Improved Chained Residual Pooling (ICRP) module as the replacement. The two separate score maps generated by MFEF1 and MFEF2 are fused with different weights to produce the fused score map, which is further handled by the Softmax function to generate the final extraction results for water and shadow areas. To evaluate the proposed framework, MRDED is trained and tested with large SAR images. To further assess the classification performance, a total of eight different classification frameworks are compared with our proposed framework. MRDED outperformed by reaching 80.12% in Pixel Accuracy (PA) and 73.88% in Intersection of Union (IoU) for water, 88% in PA and 77.11% in IoU for shadow, and 95.16% in PA and 90.49% in IoU for background classification, respectively.

## 1. Introduction 

Synthetic Aperture Radar (SAR) is an active imaging radar featuring the ability of all-day and all-weather observation. It has been broadly applied in many fields, such as military, agriculture, geo-hazards, and marine sciences [1]. SAR image classification, which identifies different types of land use and land cover, plays a pivotal role in the process of SAR image analysis. With the rapid development of remote sensing technology, the resolution of SAR images is continuously increasing; and the volume of data is getting bigger and bigger. Traditional SAR data analysis heavily relies on manually crafted features, which usually turns out to be an onerous task with huge volume of data. Therefore, it is urgent to investigate automatic procedures for SAR data processing and analysis.

In side-looking SAR or interferometric SAR (InSAR) intensity images, there are shadow areas caused by tall buildings, trees, and mountains. They are visualized as dark areas since no signal has been backscattered to the radar. In addition, many water areas on the surface of the Earth also display as very low intensity areas or nearly dark areas, since the reflection of water bodies is very similar to that of mirrors. The extraction of water and shadow areas needs to be addressed in many fields, such as flooding water evaluation [2], surface water detection [3], and building change detection [4]. Furthermore, water and shadow areas in SAR and InSAR intensity images are usually noisy, which hinder related applications, such as road extraction [5], InSAR phase filtering [6], phase unwrapping [7], Digital Elevation Model generation [8], and time-series analysis [9]. Therefore, accurate extraction of water and shadow areas, which is quite challenging to perform automatically at present with high accuracy. plays a pivotal role in SAR or InSAR image analysis. Meanwhile, deep learning has developed rapidly in recent years [10], which has been used in classification for remote sensing images in numerous projects and achieved promising performance [11,12,13]. Therefore, it is appealing to explore deep learning for automatic water and shadow extraction.

To accomplish this goal, a novel framework named Multi-Resolution Dense Encoder and Decoder (MRDED) has been proposed in this paper. There are three parts of MRDED: the Gray Level Gradient Co-occurrence Matrix (GLGCM), the Encoder network, and the Decoder network. First, the GLGCM is chosen as the low-level feature extraction approach because of its superior performance in extracting texture features from SAR images. Second, Residual Network (ResNet) is used as the Encoder to extract intermediate and high-level features of the targets. At last, the Decoder is designed to fuse the features extracted by the Encoder and further generate essential features for the final extraction of water and shadow. The contributions of this paper may be summarized as follows:

(1) As far as we know, MRDED is the first deep neural network specifically designed for automatic extraction of water and shadow from SAR images. To tackle the noisy information, we integrate several well-established deep neural networks, such as Resnet, Dense Connection Network (DenseNet), and Global Convolutional Network (GCN). The enhanced accuracy in our experiment has indicated the success of our integration.
(2) The integration of Resnet, DenseNet, and multi-resolution network has enabled the design of deeper neural network for SAR image analysis. This approach has transformed more layers of neural network into better feature representation at various resolutions.
(3) To improve the performance of the Decoder, a high-level feature fusion approach has been invented. We have developed two implementations of the Multi-level Features Extraction and Fusion (MFEF) network, named MFEF1 and MFEF2, to generate separate score map. Then, these two score maps are fused with different weights to generate the final score map for water and shadow extraction. Our experiment has proved this kind of high-level feature fusion is effective for water and shadow extraction.

The rest of the paper is arranged as follows: Section 2 gives the background of the paper, which presents the-state-of-the-art of water and shadow extraction in SAR image analysis. Section 3 describes MRDED in detail, especially the building of MFEF1and MFEF2 in the Decoder network. The experiment is depicted in Section 4, and the classification results of water and shadow are compared with several existing frameworks. We discuss how to further improve the performance of MRDED in Section 5. Finally, we conclude the paper in Section 6.

## 2. Background

SAR image classification has been very critical for SAR image understanding, and its fast development has attracted considerable interests. Ranjani and Thiruvengadam [14] proposed a classification method based on the multi-level ratio of exponentially weighted means, to compute the optimal threshold of classification. However, this thresholding approach still required manual tuning, which was very challenging to set if the pixel values of different classes were close. Hou et al. [15] proposed a Markov Random Field (MRF) method to perform SAR classification, which combined contextual information and simulated annealing algorithm. However, its performance was largely affected by noise. 

Classification has been an important component for water and shadow extraction from SAR images. Hong et al. [16] proposed a thresholding method to perform water extraction using SAR images and DEM, and they found that the extraction accuracy was greatly improved compared with experiments only using SAR images. Martinis et al. [17] evaluated four water extraction methods used at the German Aerospace Center, namely Water Mask Processor, Rapid Mapping of Flooding, TSX Flood Service, and TanDEM-X Water Indication Mask processor. These four methods achieved satisfactory water extraction results, with the accuracies higher than 90%. However, they were not completely automatic and some of them required auxiliary information (e.g., DEM datasets). Xie et al. [18] proposed a supervised water extraction method for urban area studies, which has combined both shape and polarimetric features from SAR images.

There has been limited progress in shadow extraction and existing works have largely depended on feature extraction approaches. Cellier et al. [19] studied the shadow extraction using the mean shift algorithm for building reconstruction, by fusing the amplitude and coherence of SAR images. The results indicated the extraction accuracy of shadows was significantly improved by fusion. Tison et al. [20] proposed a method by minimizing an energy function for InSAR images to identify shadow areas, which achieved good performance for isolated or high buildings. However, this shadow extraction was problematic when the building was low, or the noise of the interferometric phase was high. Jahangir et al. [21] paid special attention to shadows from different overlapping objects, which were tackled using Support Vector Machine (SVM) classification with multiple perspective SAR images. Papson and Narayanan [22] adopted Expectation Maximization to perform shadow extraction and used a Hidden Markov Model to improve the boundary identification of the shadow areas from SAR images, which could achieve 75% accuracy.

Deep learning has brought great advancements in the classification of optical images, which has been extended to SAR images classification. Long et al. [23] replaced the traditional classification method by a fully convolutional network (FCN) to perform optical image classification. The employment of FCN has been a solid contribution, but the accuracy was limited, and the details of extraction (e.g., the boundary of water bodies) were poor. Chen et al. [24] and Zheng et al. [25] improved the classification accuracy by using Conditional Random Fields (CRFs), respectively. Cheng et al. [26] exploited atrous convolution to generate high-resolution feature maps, which could reduce the loss of details during the down-sampling process of the deep learning. Lin et al. [27] and Peng et al. [28] investigated codec network to achieve high classification accuracy. Chen et al. [29] proposed an all-convolutional network without using fully connected layers, which obtained an accuracy of 99% in ten-type targets of MSTAR dataset. Huang et al. [30] presented a transfer learning method by designing an assembled CNN structure to perform classification from SAR images with limited labels, which achieved better classification accuracy. Lin et al. [31] proposed the convolutional highway unit to perform classification from SAR image, which could bring the accuracy of 99% in MSTAR dataset; but the accuracy would drop to 94.7% with reduced training data. Geng et al. [32] proposed the pre-processing method to extract preliminary and intermediate features, then automatic encoder was adopted to perform SAR image classification. To reduce the influence of speckle noise, the post-processing method has been frequently explored. Zhang et al. [33] investigated a dense Depthwise separable convolution network to perform water classification, which achieved high accuracy, but only tested with small-scale images.

Although deep learning has been proved to be a promising technique for classification, there are still several limitations to be addressed before it can be used for automatic extraction of water and shadow from SAR images. First, SAR images are very different from optical images, in which speckle noise presents an additional challenge for classification. Second, the existing networks for classification need to be tailored for enhanced context integration and features extraction for water and shadow. Third, the similarity between water and shadow often leads to considerable misclassifications using convolutional neural networks. Therefore, a novel deep learning framework, called MRDED, has been proposed in this paper specifically for water and shadow extraction with high accuracy from SAR images.

## 3. Datasets Description

**Figure 1:**
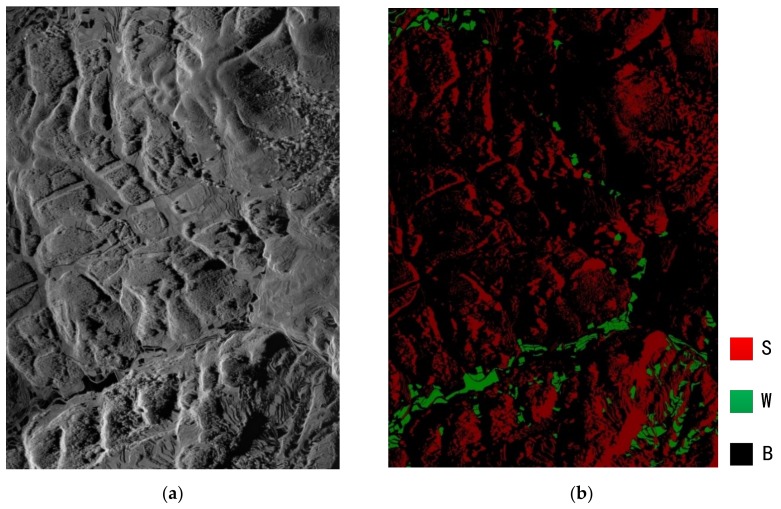
(**a**) the large-scale SAR image. (**b**) The corresponding ground truth of the SAR image. ‘S’, ‘W’, and ‘B’ denote ‘Shadow’, ‘Water’, and ‘Background’ respectively.

The datasets used in the paper are millimeter InSAR datasets acquired in Xi’an (China) in 2013, including nine large-scale SAR images with dimensions of 10240 × 13050. An example of the large-scale SAR images and the corresponding ground truth are shown in Figure 1, where the capital characters ‘S’, ‘W’, and ‘B’ denote ‘Shadow’, ‘Water’, and ‘Background’, respectively. In the experiment, only SAR images are used, and the coherence map and InSAR phase are not included. First, we use the ‘Image Labeler’ in the MatLab software to mark the three types of targets, namely, water, shadow, and background. All the shadow and water labels are checked carefully by several SAR/InSAR experts. Then, the nine-labeled large-scale images are decomposed into numerous 720 × 720 tiles, except the area selected for testing in Section 5.1. There are 1288 image tiles in total, and the ratio of training samples and validation samples is set to 4:1. 

## 4. Methodology

**Figure 2:**
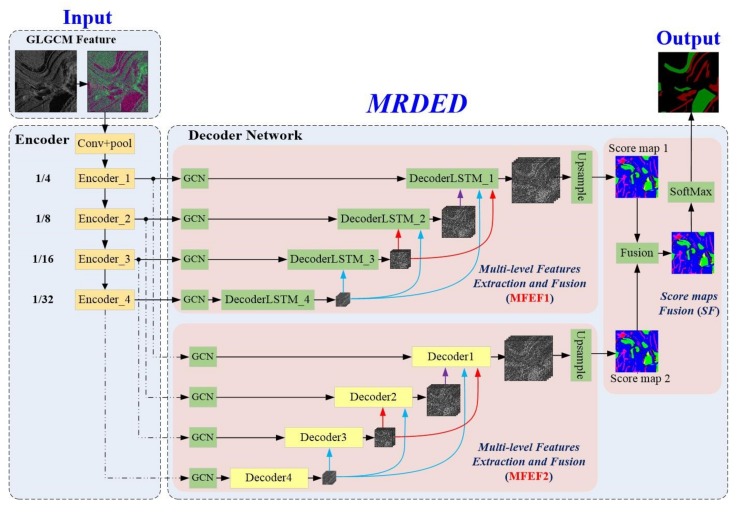
The overall architecture of the proposed network. MRDED denotes Multi-resolution Dense Encoder and Decoder framework, which contains GLGCM feature, Encoder network and Decoder network. The input is SAR images, and the output is the final extracted results of water and shadow.

The proposed Multi-Resolution Dense Encoder and Decoder (MRDED) network integrates Convolutional Neural Network (CNN) [34], Dense Connection Network (DenseNet) [35], Residual Network (ResNet) [36], Global Convolutional Networks (GCN) [28], and Convolutional Long Short-Term Memory (ConvLSTM) [37]. The overall architecture of the proposed network is delineated in Figure 2.

The proposed framework includes three parts: the Gray Level Co-occurrence Matrix (GLCM) feature extraction, the Encoder network, and the Decoder network. The GLGCM extracts low-level features, which contain detailed information for water and shadow areas. The Encoder network, based on Resnet, is used to extract intermediate and high-level features. The Decoder Networks contains two parts, the Multi-level Feature Extraction and Fusion (MFEF) part and the Score maps Fusion (SF) part. In the paper, we construct two different MFEF implementations, MFEF1 and MFEF2 (as shown in Figure 2). MFEF1 consists of four GCNs modules and four DecoderLSTM_X modules, and MFEF2 consists of four GCNs modules and four Decoder_X modules. The difference between DecoderLSTM_X and Decoder_X modules lies in ConvLSTM is employed in MFEF1 to form ICRP; but ICRP is replaced by CRP in Decoder_X. Therefore, after the multi-level features have been generated by the Encoder network, we use MFEF1 and MFEF2 to generate two separate score maps. Then, SF part is used to fuse these two score maps with different weights and new splicing method to generate the final score map. Finally, the extracted results of water and shadow are generated through the Softmax classifier.

### 4.1. GLGCM Feature

The texture is an important feature capturing the spatial structure of objects in the image, which is used extensively in image analysis and automatic classification [38]. In high-resolution SAR images, the pixels in the same types usually have the similar statistical properties which can be aggregated to acquire similar features for denoising [39]. In this paper, GLGCM is used to reduce the speckle noise of SAR images and extract the texture information.

Compared with Gray Level Co-occurrence Matrix (GLCM), GLGCM considers both grayscale information and gradient information for each pixel in the image simultaneously. The element H(i,j) of GLGCM is defined as the total number of the pixels, which has the same grayscale i in the normalized gray image and the same gradient j in the normalized gradient image. In the paper, a Sobel operator with a 3 × 3 window is used to compute the gradient value of each pixel:

(1) $$g\left(K,L\right)={\left[g_{x}^{2}+g_{y}^{2}\right]}^{1/2}$$

where K=1,2,…, M;L=1,2,…, N; M and N are the numbers of the rows and columns of the image. g(K,L) is the gradient value for the pixel of (K,L).

The normalized gradient transformation is computed as follows:

(2) $$G\left(K,L\right)=INT\left(g\left(K,L\right)\times \frac{N_{g}}{g_{M}}\right)+1$$

where INT denotes rounding operation. $g_{M}$ denotes the maximum gradient value in the image, and $N_{g}$ denotes the normalized maximum gradient value.

After grayscale normalized transformation, Equation (12) becomes:

(3) $$F\left(K,L\right)=INT\left(f\left(K,L\right)\times \frac{N_{H}}{f_{M}}\right)+1$$

where $f_{M}$ is the maximum grayscale value of the original image, and $N_{H}$ is the normalized maximum grayscale value. 

In the normalized grayscale image and gradient image, H(i,j) value can be acquired by counting the number of pixel pairs with F(m,n)=i and G(m,n)=j [38,40]. The sum of H(i,j) is:

(4) $$H=\overset{N_{H}}{\underset{i=1}{\sum }}\overset{N_{g}}{\underset{j=1}{\sum }}H_{ij}$$

Then, the normalized GLGCM is generated by the following equation:

(5) $${\hat{H}}_{ij}=H_{ij}/\left(N_{H}\times N_{g}\right)$$

where $i=1,2,\ldots ,\;N_{H},\;j=1,2,\ldots ,\;N_{g}$.

There are fifteen texture elements computed by GLGCM [40], which can be used to evaluate the relationship of grayscale and gradient between the objective pixel and the adjacent pixels, such as coherence and small gradient dominance.

**Figure 3:**
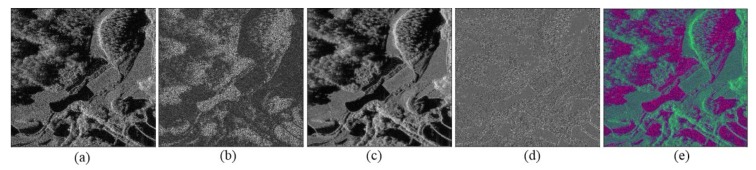
(**a**) SAR image. (**b**) Large gradient dominance. (**c**) Grayscale mean. (**d**) Coherence. (**e**) Fusion feature of (**b**), (**c**) and (**d**).

We follow [40,41,42] to heuristically select the features of large gradient dominance, grayscale mean, and correlation as low-level features to reduce the speckle noise, as shown in Figure 3. Figure 3a is the SAR image, and Figure 3b is the lager gradient dominance feature. Figure 3c is the grayscale mean, which reduces the noise in a big extent, and Figure 3d is the correlation. They extract the different texture features for better classification. Since most existing deep neural networks expect input images with three channels [43,44], so we follow this to construct the input with three channels. The three features (as shown in Figure 3b–d)) are concatenated to form a three-channel input, which is shown in Figure 3e.

### 4.2. The Encoder Network

The Encoder network is composed of ResNet with 101 layers. ResNet is used to perform image classification initially. According to the FCN proposed by Long et al. [23], the network used for classification should be changed to FCN for dense classification, if the Global Average Pooling (GAP), fully connected layer, and Softmax layer in the residual network are removed. 

#### 4.2.1. Residual Network

For image classification, there are several excellent networks, such as RefineNet [27], VGG-19 network [45], FCN [23], ResNet [36,46], and DenseNet [35]. 

**Figure 4:**
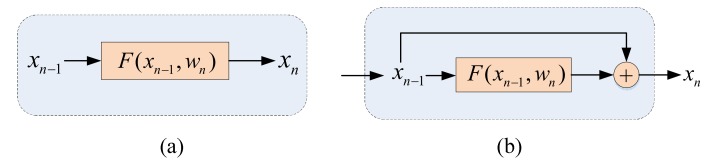
(**a**) The traditional convolutional neural network. (**b**) Residual unit.

In traditional CNN, the network is simply stacked one layer by another, as shown in Figure 4a. We assume that the function of any layer in the network is expressed as F, then the output $x_{n}$ of the nth layer can be represented as:

(6) $$x_{n}=F\left(x_{n-1},w_{n}\right)$$

where $x_{n-1}$, $x_{n}$, and $w_{n}$ are the input, output, and the weight of the n-th layer. 

He et al. [36,46] has observed that the performance of network would not be improved by simply stacking layers, which might even decrease when the number of layers was too deep. Therefore, they have presented Residual network (ResNet), which made CNN break through the limitation of layers depth and achieved better feature representation within deeper layers. The ResNet is stacked by a series of residual units, which is shown in Figure 4b. Instead of calculating the output $x_{n}$ directly, each residual unit calculates a residual and then adds it to the input $x_{n-1}$:

(7) $$x_{n}=x_{n-1}+F\left(x_{n-1},w_{n}\right)$$

Using Equation (7) recursively, He et al. [46] has demonstrated the output of the mth residual unit as:

(8) $$x_{m}=x_{n}+\overset{m-1}{\underset{i=n}{\sum }}F\left(x_{i},w_{i+1}\right)$$

The above equation shows that the network has good backpropagation performance. Assuming the Loss function of the network is l, the following relation can be obtained according to the backward chain rule [47]:

(9) $$\frac{\partial l}{\partial x_{n}}=\frac{\partial l}{\partial x_{m}}\frac{\partial x_{m}}{\partial x_{n}}=\frac{\partial l}{\partial x_{m}}\left(1+\frac{\partial }{\partial x_{n}}\overset{m-1}{\underset{i=n}{\sum }}F\left(x_{i},w_{i+1}\right)\right)$$

(10) $$\frac{\partial l}{\partial w_{n}}=\frac{\partial l}{\partial x_{n}}\frac{\partial x_{n}}{\partial w_{n}}=\frac{\partial x_{n}}{\partial w_{n}}\left(\frac{\partial l}{\partial x_{m}}+\frac{\partial l}{\partial x_{m}}\overset{m-1}{\underset{i=n}{\sum }}\frac{F\left(x_{i},w_{i+1}\right)}{\partial x_{n}}\right)$$

The update of the weights depends on $\frac{\partial l}{\partial x_{m}}$ and $\frac{\partial l}{\partial x_{m}}\sum_{i=n}^{m-1}\frac{F\left(x_{i},w_{i+1}\right)}{\partial x_{n}}$. Only the latter depends on the depth of the network, not the former. Meanwhile, there will be no gradient disappearance if their sum is not zero. This is rarely the case, so the gradient can be smoothly transmitted from the upper layers of the network to the lower layers, enabling the training of deeper networks.

#### 4.2.2. The Structure of the Encoder Network

The specific structure of the Encoder network is shown in Table 1. In the matrix of Conv2_x of Table 1, 1×1 and 64 denote the size and number of the convolutional kernel, and 3×3 means the number of the residual convolutional unit, which contains three convolutional layers. The 101-layer refers to the number of the weight layers in the network, and the specific calculation method is (3 + 4 + 23 + 3) × 3 + 1 + 1 =101, without the fully connected layer. In Table 1, the initial value of the image for Output size is assumed to be 512 × 512.

### 4.3. The Decoder Network

The internal structure of Decoder network is shown in Figure 2. It contains two MFEF networks, and each of them includes four Global Convolutional Networks (GCNs), four DecoderLSTM_X (X = 1, 2, 3, 4) modules or four Decoder_X (X = 1, 2, 3, 4) modules, and the Dense connections.

#### 4.3.1. Global Convolutional Network

**Figure 5:**
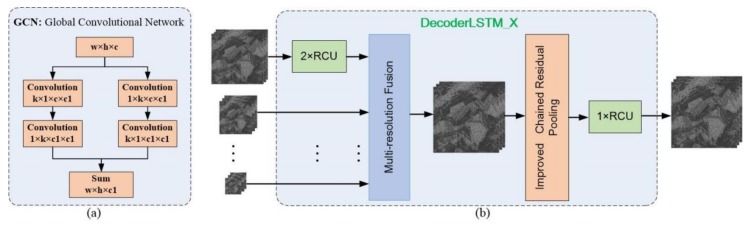
(**a**) The GCN network. (**b**) The network of DecoderLSTM_X.

GCN is composed of two separable convolutions [48], 1×k+k×1 and k×1+1×k, which is shown in Figure 5a. GCN can implement dense connections in a k×k region of the input feature map, and its calculations and parameters are only O(2/k) compared with the ordinary convolutional kernel. GCN performs dense connection [28] in the large receptive field in the network, which can improve the accuracy the classification results and has the function of dimension matching.

#### 4.3.2. DecoderLSTM_X Module

The four DecoderLSTM_X (X = 1,2,3,4) modules have the same internal structures with different parameters. This module consists of three parts (as shown in Figure 5b), namely, Residual Convolution Unit (RCU), Multi-Resolution Fusion (MRF), and Improved Chained Residual Pooling (ICRP). In the Decoder Network, the DecoderLSTM_X has multiple inputs, including high-resolution features generated from the Encoder network and low-resolution semantic features output by the DecoderLSTM_Y (Y > X). The feature output by the Encoder network is processed by two RCU units and then fused with low-resolution semantic features. Then the fused features are processed further by the ICRP module to extract new semantic features, which will be tuned by an RCU unit and then they will be output to the higher-resolution decoder module (e.g., DecoderLSTM_X (X = 2, 3, 4)) or processed to generate the score map (DecoderLSTM_1). Where DecoderLSTM_4 has only one input from the feature generated by the Encoder. 

##### (A) Residual Convolution Unit 

**Figure 6:**
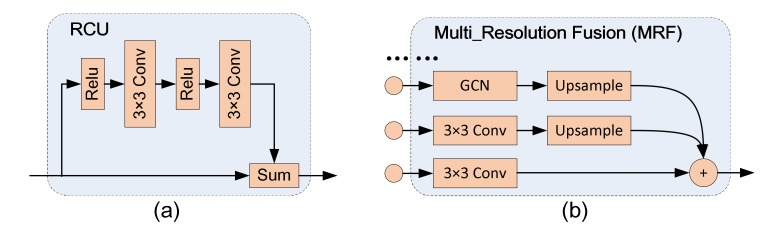
(**a**) the structure of RCU. (**b**) the structure of MRF.

The Residual Convolution Unit (RCU) consists of the residual convolution unit [27] without the Batch Normalization (BN) layer. The main functionality of RCU is to fine-tune the weight of the pre-trained residual network model, and meanwhile, it can adjust the input features for the following processing. The structure of RCU is shown in Figure 6a, which includes two Relu layers and two 3 × 3 convolutional layers.

##### (B) Multi-Resolution Fusion

The Multi-Resolution Fusion (MRF) module is shown in Figure 6b, whose functionality is to fuse the features generated by the Encoder and the features output by the Decoder module with higher-level. Assuming *H*, *W*, and *C* are the height, width, and channel number of the input feature map, then their consistency needs to be guaranteed before the fusion of features with different resolutions. First, the channel number *C* of the input features from the current Encoder and the previous Decoder module is unified by a convolution operation, and if there are dense connected inputs, the dimension matching should be performed through the Global Convolutional Network (GCN). Then, all low-resolution feature maps are upsampled to high-resolution feature map to ensure the uniform of H×W. 

##### (C) Improved Chained Residual Pooling 

The Chained Residual Pooling (CRP) developed by Lin et al. [27] can be used to extract the contextual information from the background in a large image area, playing an important role in the Decoder module. It is also used in [49], and the only difference of CRP is that CONCAT operation is used to fuse the pooled features instead of SUM used in RefineNet [27]. In this paper, we introduce Convolutional Long Short-Term Memory (ConvLSTM) into the CRP to simultaneously handle spatio-temporal sequences.

• Convolutional Long Short-Term Memory

Long Short-Term Memory (LSTM) [50] is a type of Recurrent Neural Network (RNN) [51]. Its internal memory units could store and process the information across a wide range of time, which help solve the problem of gradient disappearance in RNN. LSTM has been widely used in various fields. Visin et al. [52] has proposed the Renet network for image classification. It is completely built by LSTM, which is a very meaningful attempt though the result is slightly worse than the network built by convolution and pooling. Li et al. [37] has presented LSTM-CF network for image classification. It inserts several LSTM layers into the CNN to extract a broad range of dependence relation among pixels, which gains good classification performance compared to the local receptive field of the convolution network. However, the vector conversion required by LSTM has always incurred considerable spatial structure information loss, since LSTM is based on temporal sequential data, not spatial data. Therefore, Shi et al. [53] has developed the Convolutional LSTM (ConvLSTM) network to solve this problem by introducing a convolutional structure into LSTM, which could simultaneously resolve spatio-temporal sequences.

In addition to introducing convolution operations and higher dimensions of data representation, ConvLSTM has a similar structure to LSTM. The main innovation of LSTM is flexible memory unit, which can change the information in the unit through input gates, output gates, and forgetting gates. The forgetting gate and the input gate separately determine how much information will be discarded from the memory unit and how much information will be input into the unit, and the output gate determines how much information will be output based on the current unit. 

Suppose the inputs are $X_{1},\cdots ,\;X_{t}$, and the memory units are $C_{1},\cdots ,\;C_{t}$. The status of the hidden layer is $H_{1},\cdots ,\;H_{t}$, and the input gate, the forgetting gate, and the output gate are $i_{t},f_{t},\;O_{t}$, respectively. These variables are all 3-D tensors. The symbol ’*’ indicates the convolution operation and ’°’ indicates the Hadamard product:

(11) $$i_{t}=\sigma \left(W_{xi}*X_{t}+W_{hi}*H_{t-1}+b_{i}\right)$$

(12) $$f_{t}=\sigma \left(W_{xf}*X_{t}+W_{hf}*H_{t-1}+b_{f}\right)$$

(13) $$O_{t}=\sigma \left(W_{xo}*X_{t}+W_{ho}*H_{t-1}+b_{o}\right)$$

(14) $$C_{t}=f_{t}\;{}^{\circ}\;C_{t-1}+i_{t}\;{}^{\circ}\;tanh\left(W_{xo}*X_{t}+W_{hc}*H_{t-1}+b_{c}\right)$$

(15) $$H_{t}=O_{t}\;{}^{\circ}tanh\left(C_{t}\right)$$

where σ denotes Sigmoid function, and *W*_*x*~_ and *W*_*h*~_ are the 2-D convolutional kernels.

**Figure 7:**
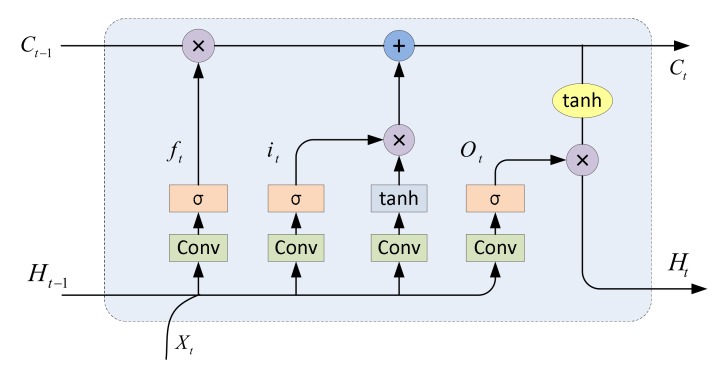
The structure of ConvLSTM. The new memory $C_{t}$ and output $H_{t}$ will be generated by updating the internal memory $C_{t-1}$ according to the current input $X_{t}$ and the previous output $H_{t-1}$.

The specific structure of ConvLSTM is shown in Figure 7.

• The Improved Chained Residual Pooling module

**Figure 8:**
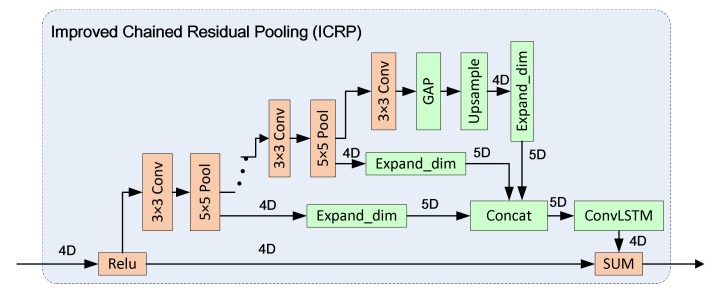
The structure of Improved Chained Residual Pooling (ICRP). 4D and 5D denote 4-dimensional tensor and 5-dimensional tensor respectively.

The proposed Improved Chained Residual Pooling (ICRP) module is shown in Figure 8, in which ConvLSTM is employed.

ICRP has been improved in three aspects compared with CRP. First, we swap the position of convolution layer and the pooling layer, which are implemented as a 5 × 5 maximum pooling layer follows a 3 × 3 convolution layer; and meanwhile the number of the convolution and pooling modules is increased to four. Second, a Global Average Pooling (GAP) is added to obtain the global information of the feature map. As shown in Figure 8, the biggest advantage of CRP is that it can continuously pool the input feature map, so a 5 × 5 window can be used to obtain a wide range of contextual information. Third, ConvLSTM unit is used to integrate the output features of each pooling module. At present, in the network of CRP, the fusion of feature maps in different pooling stages mostly uses SUM [27] or CONCAT [49]. It can be seen from CRP structure [27] that the features output by the previous pooling module will be utilized by the latter pooling module. The difference between the neighboring features is several times of pooling, so there is a certain temporal and spatial correlation among these features. The spatial operation by simple SUM or CONCAT is not enough to generate semantically distinctive features, so ConvLSTM is introduced to integrate the spatial and temporal information of features. ConvLSTM requires the input features to be 5D tensors (i.e., [samples, time, rows, cols, channels]), but the features in CRP are 4D tensors (i.e., [samples, rows, cols, channels]), so we need to increase their dimensionality. As shown in Figure 8, we perform a dimension expansion to features output by each pooling module, connect four features along the time dimension, and bring the generated features to the ConvLSTM unit for further processing to output a 4D feature. We set time for the four feature maps in CRP, namely, $t_{1}$, $t_{2}$, $t_{3}$, and $t_{4}$. we first feed the feature of the moment $t_{1}$ into ConvLSTM, which will generate a memory state $C_{t_{1}}$ of the current feature with a hidden layer state $H_{t_{1}}$. When the moment $t_{2}$ comes, ConvLSTM will decide how much previous information to forget and how much new information to add based on the current input and the state of the previous moment. Using such loops, a new feature will be output according to the live memory state. The ICRP can extract better contextual information than CRP, allowing the network to accommodate higher resolution images, and LSTM could also tackle the problem of gradient disappearance.

#### 4.3.3. Decoder X Module

**Figure 9:**
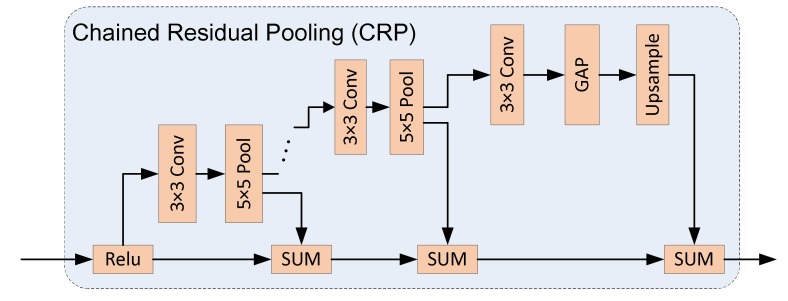
The structure of the Chained Residual Pooling (CRP).

There are two Decoder Modules used in our Decoder Network. One is the DecoderLSTM_X which is introduced above. Another is the Decoder_X module. The difference between them is that ConvLSTM is not used in Decoder_X module. Instead of using ICRP, we implement Chained Residual Pooling (CRP), which is shown in Figure 9. It consists of series of 3 × 3 Convolution layers and 5 × 5 Pooling layers. Then GAP, upsample, and SUM are used to generate the high-level features, based on traditional CRP [27].

#### 4.3.4. Dense Connection

DenseNet [35] has developed the idea of shorter connections based on the ResNet and has introduced more connections among layers. DenseNet could further mitigate the problem of gradient disappearance and enhance the propagation and reusability of features. Therefore, we follow DenseNet to perform dense connections in the paper. It means that the output of DecoderLSTM_X or Decoder_X (X = 1, 2, 3, 4) is passed to each DecoderLSTM_Y or Decoder_Y, where Y < X (as shown in Figure 2). This allows each DecoderLSTM_X or Decoder_X to use all the previous high-level features, and the features are reused to correct errors from previous Encoder_X (X = 1, 2, 3, 4). The dense connection can effectively fuse features at different resolutions, and the gradient during training can be smoothly transmitted among various decoding modules, alleviating the problem of gradient disappearance.

#### 4.3.5. Score Map Fusion

The score map fusion performs fusion of the two score maps generated by MFEF1 and MFEF2 with different weights and the new splicing method. The specific experiments of MFEF1 and MFEF2 are described in Appendix A. The weights have been determined heuristically, and the new splicing method is introduced as follows.

SAR images used in the paper are all large-scale images, while deep learning requires small-scale input images for training. Therefore, we generally decompose the large-scale image into small-scale images. However, when we cut the original image, we may encounter a situation where a large water body is divided into two or more parts, which undermines the integrity of the target. Although the results can be generated by stitching small images directly, there will be distortion at the edge areas.

To address the problem above, a new splicing method is presented in the paper inspired by Xing and Sieber [54] to reduce or eliminate the errors at the splicing. First, we use a sliding window with the size of 512 × 512 to cut the large-scale image with a stride of 256. It can minimize the probability of object cutting in input images and repeat testing for almost all the areas of the image to reduce the probability of misclassification. Then, the splicing of testing results is performed. In this process, the score map matrixes output by the network are used instead of the classified images. For the overlapping regions of score maps, weighted averaging is employed to reduce splicing errors. By this means, all processed areas are spliced into one whole score map, which is fed to the Softmax layer to generate the classification probability of each position and the index map is finally produced.

## 5. Experiments and Results

### 5.1. Study Area

**Figure 10:**
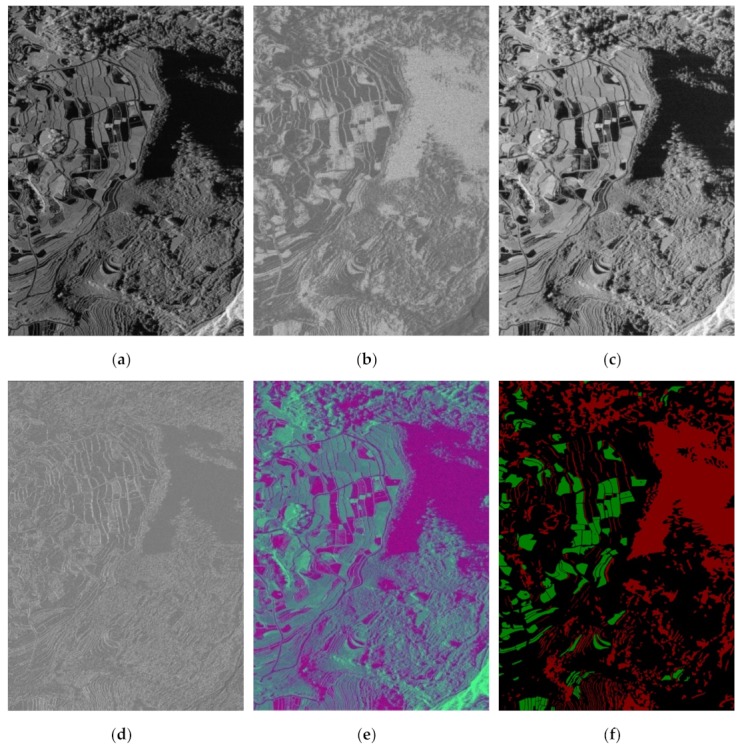
The SAR image, features and ground truth. (**a**) SAR image. (**b**) Large gradient dominant feature. (**c**) The grayscale mean feature. (**d**) The correlation feature. (**e**) The fusion feature of (**a**), (**b**) and (**c**). (**f**) The ground truth, the red color, green color, and black color denote shadow, water, and background respectively.

In the experiment, an area of a large-scale SAR image with a size of 4096 × 4608 is tested using the trained model. The SAR image is shown in Figure 10a. In the experiment, we get the large gradient dominance, the grayscale mean feature, the correlation feature, and the fusion feature for the SAR image, which are shown in Figure 10b–e respectively. According to Figure 10e, the water and shadow areas are different from other targets. Figure 10f shows the ground truth of the SAR image.

### 5.2. Adjustment of the Number of Feature Maps

The most time-consuming part of deep neural work is the training stage, which is significantly influenced by the depth of the network, the number of parameters, and the number of feature maps that need to be calculated. The size of the output of the 101-layer residual network is 256, 512, 1024, and 2048. There are two advantages to do so: one is to reduce the utilization of memory; while the other is to save the training time.

To address problems above, four sets of comparative experiments are carried out using the dataset. The number of the feature maps is set to 1, 1/2, 1/4, and 1/8 of the original RefineNet respectively. Individual results are shown in Table 2. The time in Table 2 refers to the average time to perform one subset training [55], which contains 200 images. We set the batch size to 1 due to the limited memory on the Graphic Processing Unit (GPU), and we employ subsets for better organization in the training. The accuracy is the accuracy of the validation set, and the evaluation criterion used is the Mean Intersection over Union (MIoU) [56,57]. MIoU is the most commonly used evaluation criterion for deep learning-based classification. It calculates the ratio of the intersection and the union between the segmentation results and the labels. The specific calculation formula is as follows:

(16) $$MIoU=\frac{1}{k+1}\overset{k}{\underset{i=0}{\sum }}\frac{p_{ii}}{\sum_{j=0}^{k}p_{ij}+\sum_{j=0}^{k}(p_{ji}-p_{ii})}$$

where $p_{ii}$ is the sum of the pixels belonging to the type i both in the label and the classification result, and $p_{ij}$ is the sum of the pixels which are type i in the label but type j in the classification result. k is the number of classes (*k* = 2 in the paper, which stands for water and shadow). The other targets are marked as the background. Therefore, there are three categories in total. 

According to the experiment results, when the numbers of the feature maps input into the Decoder are 64, 64, 64, and 128, the MIoU of the validating set is only reduced by 0.1%, but the training time is nearly half less than the original setting (256, 256, 256, and 512). Therefore, this configuration of feature maps (64, 64, 64, and 128) are used to train the network in the study.

### 5.3. Setting of Training Parameters and Data Enhancement

During the training of the network, the learning rate and the weight decay are set to $e^{-5}$ and 0.995 respectively. For GCN module between the Encoder module and the Decoder module, the size of the convolution kernel is set to k=9, and c1 is 64, 64, 64, and 128, respectively. In the Multi-Resolution Fusion (MRF) module, the setting of the GCN parameter k is the same as before, and c1 is adjusted according to the dimension of the input high-resolution feature from the encoder module. In the ConvLSTM, the size of convolutional kernel is set to 3 × 3, and the stride is set to 1. During the training, some traditional data enhancement operations are also adopted to process the dataset, such as horizontal and vertical mirroring, and random cropping with a window size of 512 × 512.

### 5.4. Results and Analysis

A total of two sets of comparative experiments have been done to validate the performance of the proposed framework. The first set of experiment is to test the proposed framework with different network configurations, such as **Encoder_MFEF2**, **Encoder_MFEF1**, **GLGCM_Encoder_MFEF1**, **GLGCM_Encoder_MFEF1_2**, and **Lee_Encoder_MFEF1**. All these bold ones represent the corresponding frameworks. **Encoder_MFEF2** or **Encoder_MFEF1** denotes it only uses the framework combining the Encoder network and the MFEF2 network or MFEF1 network in Figure 1 to perform classification for SAR images, which we use to compare their abilities of classification. **GLGCM_Encoder_MFEF1** denotes it uses the same framework as **Encoder_MFEF1** to perform classification by adding GLGCM features of the SAR image. **GLGCM_Encoder_MFEF1_2** uses the same framework as **GLGCM_Encoder_MFEF1**, but the final classification result is generated by the new splicing method proposed in Section 4.3.5. **Lee_Encoder_MFEF1** uses the same framework as **Encoder_MFEF1** to perform classification for 5 × 5 Lee-filtered [58] SAR images. The proposed network in the paper (**MRDED**, as shown in Figure 1) also adopts the new splicing method in Section 4.3.5. While the second set of experiment is mainly to compare the proposed framework with several other existing and main stream deep learning frameworks in SAR images classification, such as FCN-8S [23], the RefineNet [27], Large_Kernel_Matters [28], and ResNet-101_FCN [36]. 

The classification accuracy (testing accuracy) of the first set of experiment is shown in Table 3. ‘PA’ means pixel accuracy, namely, the percentage of the correctly classified pixels to the total pixels, which is computed by Equation (17). ‘MPA’ denotes Mean Pixel Accuracy, which can be computed by Equation (18):

(17) $$PA=\frac{\sum_{i=0}^{k}p_{ii}}{\sum_{i=0}^{k}\sum_{j=0}^{k}p_{ij}}$$

(18) $$MPA=\frac{1}{k+1}\sum_{i=0}^{k}\frac{p_{ii}}{\sum_{j=0}^{k}p_{ij}}$$

where $p_{ij}$ denotes the number of pixels that are type i but are classified as type j, and k+1 is the total number of the types. 

From Table 3, we can see the proposed framework **Encoder_MFEF2** could reach the PA of 0.9101 and MIoU of 0.7651. The proposed framework **Encoder_MFEF1** could acquire better PA than **Encoder_MFEF2**, which indicates the introduced ConvLSTM can improve the classification, but the improvement is not very big. **Lee_Encoder_MFEF1** gives the worst classification accuracy of these framework. That may be because the Signal and Noise Ratio (SNR) of the SAR image is very good, the detailed information of the targets is destroyed when using Lee filter again. The PA of **GLGCM_Encoder_MFEF1** is 0.41% less accurate than **Encoder_MFEF1**, but its MPA and MIoU are 0.56% and 0.6% higher respectively. Which proves that GLGCM is good for improving classification accuracy. The PA and MIoU of **GLGCM_Encoder_MFEF1_2** are 1.02% and 1.59% higher respectively than **GLGCM_Encoder_MFEF1**, which proves the effectiveness of the new splicing method. The proposed framework MRDED obtains the best performance, suggesting that MRDED is a powerful framework for water and shadow classification. 

**Figure 11:**
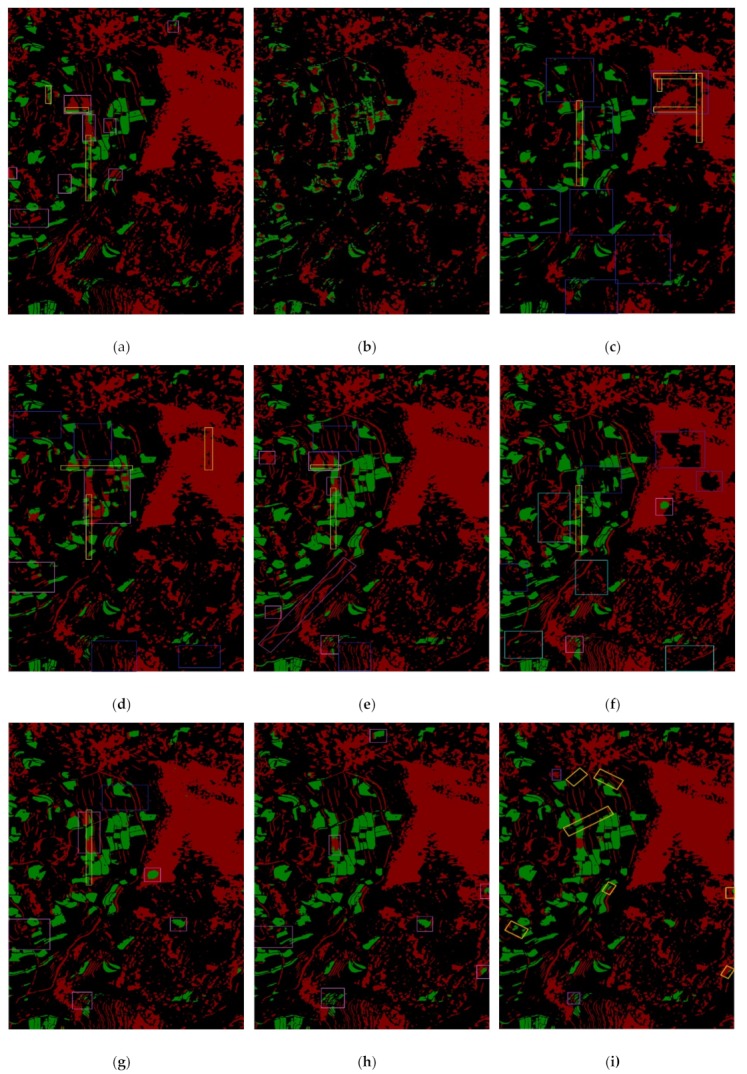

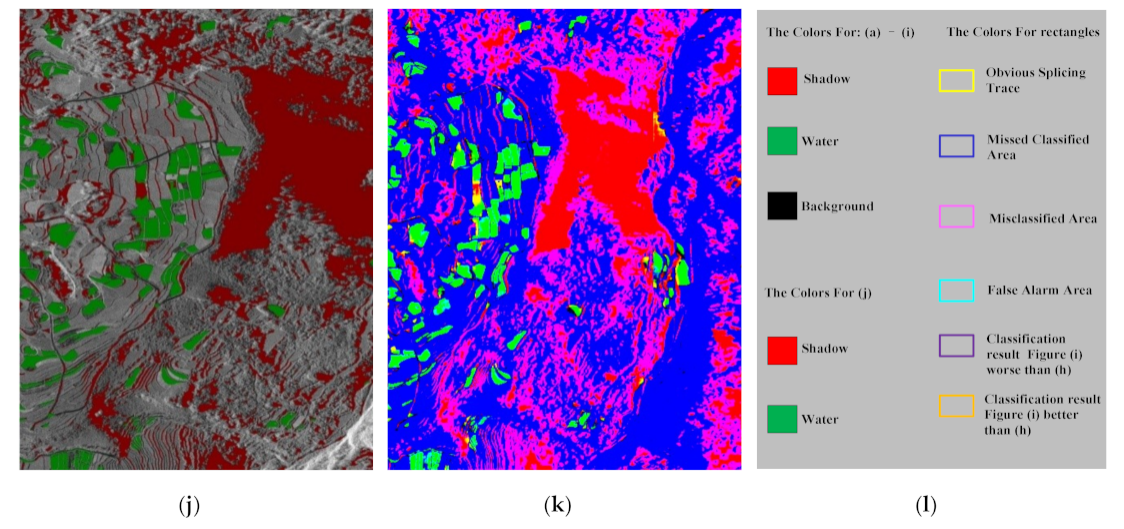
The extracted results for water and shadow from the SAR image. (**a**) The extracted results by **RefineNet**. (**b**) the extracted results by **FCN-8s**. (**c**) The extracted results by **ResNet-101_FCN**. (**d**) The extracted results by **Large_Kernel_Matters**. (**e**) The extracted result by **Encoder_MFEF1**. (**f**) The extracted result by **Lee_Encoder_MFEF1**. (**g**) The extracted result by **GLGCM_Encoder_MFEF1**. (**h**) The extracted result by **GLGCM_Encoder_MFEF1_2**. (**i**) The extracted result by **MRDED**. (**j**) The fusion map of SAR image and (**i**). (**k**) The visualization result of the results by **MRDED**. (**l**) Colors description for the classification results from (**a**) to (**j**).

To further analyze the classification performance of the proposed framework, the second set of experiment gives a comparison between MRDED and other classification frameworks. The classification results are shown in Figure 11, and the accuracy of the frameworks is shown in Table 4. For simplicity, we use **EN_M1**, **L_En_M1**, **R-101_FCN, G_En_M1**, **G_En_M1_2**, and **L_K_M** to represent **Encoder_MFEF1**, **Lee_Encoder_MFEF1**, **ResNet-101_FCN, GLGCM_Encoder_MFEF1**, **GLGCM_Encoder_MFEF1_2,** and **Large_Kernel_Matters** respectively.

According to Figure 11a, we find there are many misclassified areas compared with the ground truth (as shown in Figure 10f), such as the areas inside the pink rectangles. In these areas, lots of water bodies are misclassified as shadow. Therefore, the classification accuracy for water is lower, only with a PA of 0.6276. The classification accuracy for shadow is higher, with a PA of 0.8829. Therefore, the classification performance of **RefineNet** for water and shadow from SAR image is not good. In addition, we can see obvious boundary lines at the splicing, which are marked with yellow rectangles. It indicates that it is easy to generate misclassification at the slicing.

Figure 11b–d display the classification result by **FCN-8s**, **ResNet-101_FCN**, and **Large_Kernel_Matters** respectively from the SAR image. In original **FCN-8s** framework, VGG-19 network is used to extract features, while **ResNet-101_FCN, Large_Kernel_Matters,** and the frameworks proposed in the paper all use ResNet-101 network. Therefore, for easy and fair comparison of the algorithms, ResNet-101 network is used in **ResNet-101_FCN** framework instead of VGG-19 network. We find in Figure 11b, there are many missed detection and false detection areas for water and shadow. From Table 4, we could also see the PAs for water and shadow are very low, and the MIoU is only 0.6166. However, while using ResNet-101 network to replace VGG-19 network, the classification accuracy is improved significantly, especially for water extraction, with the improvement from 0.4011 to 0.5769. The corresponding results in Figure 11c also clearly show this. We find that it does not have much improvement in the classification accuracy of the shadow areas, and there are still many missed detection areas (the blue rectangles in Figure 11c). Compared with VGG-19, ResNet-101 could extract more distinguishing features in the experiment. While for **Large_Kernel_Matters**, as a codec network, it does not directly fuse the multi-layer features extracted by the basic network (VGG-19) but uses the decoding network to process the features generated by the coding network. It achieves much better classification result than **FCN-8s** and **ResNet-101_FCN** in shadow, and a less accuracy than **ResNet-101_FCN** in the tern of water and background, which could be seen in Figure 11d and Table 4. However, there are still some obvious missed areas and misclassified areas. According to the comparable experiment for the extraction of water and shadow using the three frameworks, we find a good coding network can extract more distinctive features, and a good decoding network can further fuse and process features generated by the encoding network for better classification results.

While using the first framework **Encoder_MFEF1** proposed in the paper, it achieves much better classification result than all the four previous frameworks (**RefineNet**, **FCN-8s**, **ResNet-101_FCN**, and **Large_Kernel_Matters**) used in the paper, which is shown in Figure 11e and Table 4. It produces better detailed information and reduces the misclassification between water and shadow. Compared with Figure 11c,d the splicing problem is also solved. Therefore, the introduced ConvLSTM could improve the performance of the framework, and finally increases the classification accuracy. According to Table 4, the PAs of water and shadow are 0.7147 and 0.8955, respectively, and MIoU is 0.7699, which indicates the proposed framework **Encoder_MFEF1** is effective in improving classification accuracy for water and shadow from SAR images. Nevertheless, there are still several misclassified areas (the pink rectangles) and missed classified areas (the blue rectangles). For the framework of **Lee_Encoder_MFEF1**, according to Figure 11f, we find that lots of water and shadow areas are not detected (the blue rectangles), and many false alarm areas exist (the cyan-blue rectangles). The classification accuracies and IoU are all reduced compared with the framework of **Encoder_MFEF1** according to Table 4, Which indicates that simply filtering the SAR image and discarding the detailed information of the image would reduce the accuracy of the final classification result. 

By integrating the GLGCM features of the SAR image into the framework of **Encoder_MFEF1**, we get the framework of **GLGCM_Encoder_MFEF1**, the classification of which is shown in Figure 11g. We find that the classification accuracy of water is better than **Encoder_MFEF1** according to Table 4, probably improved by 6%, and the accuracies of shadow and background are slightly lower, but the MPA and MIoU both increases. Which proves the effect of extracting texture features with GLGCM to improve classification accuracy. When the new splicing method (Section 4.3.5) is introduced, we get the framework of **GLGCM_Encoder_MFEF1_2**, the classification result is shown in Figure 11h. All the classified results of the previous frameworks have the obvious splicing problem, but it is solved in this framework. In Figure 11h, there is almost no splicing problem, and the final classification result is improved too. Though the PA is a little lower than the framework of **G_En_M1**, but its IoU is higher, and the accuracy of shadow is greatly improved. The final MIoU is increased nearly by 1.6%. However, we find there are still several misclassified areas and the PA of water is only 75.73%.

The classification result of **MRDED** is shown in Figure 11i. For **MRDED**, the weights of 0.7 and 0.3 are used for the fusion of the two score maps generated by MFEF2 and MFEF1 respectively. Because we find through numerous experiments that MFEF2 could achieve slightly better classification results for water than MFEF1, though MFEF1 could achieve much better overall classification for the three types of targets. Through many experiments, we select the weight of 0.7 for MFEF2 and the weight of 0.3 for MFEF1. As a result, we find that **MRDED** achieves better classification results for water and shadow in many orange rectangles areas than **GLGCM_Encoder_MFEF1_2**, but there are several purple rectangles areas with a slightly worse classification result. The accuracy for water extraction is greatly improved, nearly by 4.4% in PA and 3% in IoU. The accuracy for shadow is reduced by 1.95% in PA, but its IoU is increased by 0.62%. The final MPA and MIoU are improved by 1.09% and 1.3% respectively, which prove that **MRDED** framework is the best framework in auto-extraction of water and shadow from SAR images.

**Figure 12:**
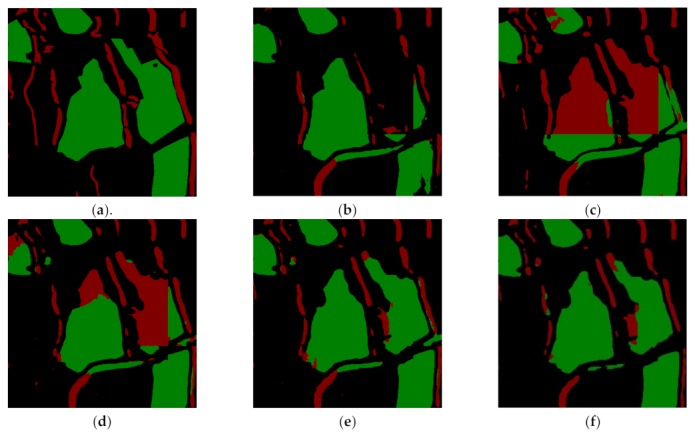
Local segmentation results of different networks proposed in this paper. (**a**) The ground truth. (**b**) **Lee_Encoder_MFEF1** result. (**c**) **Encoder_MFEF1** result. (**d**) **GLGCM_Encoder_MFEF1** result. (**e**) **GLGCM_Encoder_MFEF1_2** result. (**f**) **MRDED** result. The red color and green color denote shadow and water respectively.

For more detailed analysis of the classification performance with splicing, Figure 12 shows local enlargements of the classification result generated by different frameworks. We find there are obvious splicing phenomenon, misclassification or missed classification in Figure 12 b–d, because we fuse the classification results by directly splicing. However, when we use the new splicing method (Section 4.3.5), the splicing phenomenon disappears, and misclassification is reduced significantly in Figure 12e, while in Figure 12f, the proposed framework further improves the classification accuracy for water and shadows. 

**Figure 13:**
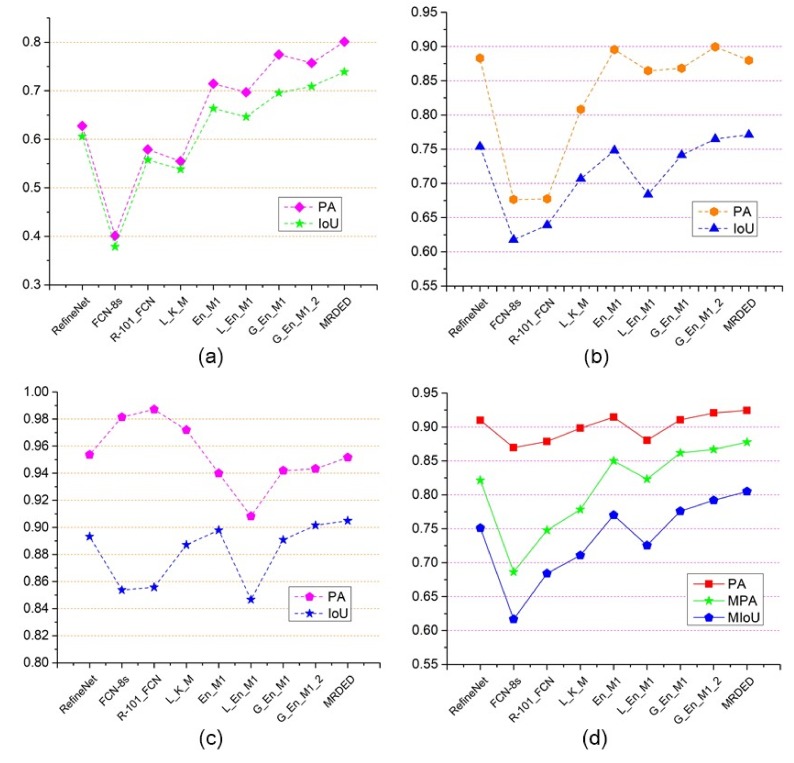
The accuracies for different types of targets with different frameworks. (**a**) The classification accuracy for water with different frameworks. (**b**) The classification accuracy for shadow with different frameworks. (**c**) The classification accuracy for background with different frameworks. (**d**) The overall accuracy for the three targets.

In order to understand the classification performance of various frameworks more intuitively, accuracies for the same type of target with different frameworks have been given in Figure 13 according to Table 4. The abscissa axis represents the nine types of frameworks, and the ordinate axis represents the accuracy of the same type of target. From Figure 13a, for water classification, we find the worst classification performance is the framework of **FCN-8s,** only with PA and IoU of nearly 0.4, and the best classification effect is the framework of **MRDED**, with PA of 0.8 and IoU of nearly 0.74. For shadow classification, according to Figure 13b, the frameworks of **EN_M1** and **G_EN_M1_2** achieve better classification results than the other frameworks, but **G_EN_M1_2** has higher IoU than **EN_M1** because of the introduction of new splicing method and the GLGCM features. The proposed framework **MRDED** and the framework of RefineNet achieve slightly lower PA than **EN_M1** and **G_EN_M1_2**, but the **MRDED** has higher IoU than the other frameworks, which proves it is a more reliable framework for shadow classification. For background classification, according to Figure 13c, **FCN-8s** and **R-101_FCN** achieve in PA of over 98%, but their IoUs are very low, which show there are many false alarm areas for background classification. Though the classification accuracy of **MRDED** is not the highest (only 95%), but its IoU is quite satisfying, which indicates its false alarm areas for background classification are the best. Figure 13d shows the overall accuracy of the three types of targets, including PA, MPA, and MIoU. From which we find, **FCN-8s** has the worst classification performance, and the proposed framework of **MRDED** has the best classification results. The final PA, MPA, and MIoU of **MRDED** could reach 0.9244, 0.8776, and 0.8049, respectively. 

In the paper, MRDED, a new framework specifically designed for water and shadow extraction from SAR images has been presented. It has achieved enhanced performance in our experiment. However, there are still several problems that need to be further explored. The first one is the optimization of weights to fuse the two score maps generated by MFEF1 and MFEF2 modules. In the paper, we give the weights of 0.7 for MFEF2 and 0.3 for MFEF1, respectively, following experiments, but how to optimize their values automatically still remains an open challenge, which will be a focus of our future work. The second one is how to further improve the classification performance of the MRDED. Comparing Figure 11i with the ground truth (Figure 10f), we find some water areas and roads are misclassified as shadows; and some shadow areas are classified as background or water. Therefore, we plan to investigate the scattering characteristics of water and shadow in SAR images in the future, to further ameliorate the classification accuracy. Third, the accuracy of water extraction is not very high (80.12%), since we have limited number of water samples in our experiment. This needs to be trained with SAR images covering larger water bodies. The last problem is the boundary of water and shadow extraction. As shown in Figure 12, we find the boundary extraction is not very satisfying with ground-truthing, especially the missing of details. Thus, we will consider other features for detailed boundary representation, especially some segmentation methods to improve the extraction results of water and shadow.

## 6. Conclusions

In the paper, a new framework, MRDED, has been presented to perform the extraction of water and shadow from large-scale SAR images. It contains three parts, namely, the GLGCM, the Encoder network, and the Decoder network. The GLGCM extracts low-level features from SAR images. The Encoder network is based on the ResNet to extract features with different resolutions. The proposed Decoder network includes two parts, called MFEF and SF, respectively. The MFEF performs the extraction and fusion of the features to generate the score map for classification. In this paper, two MFEF modules are proposed and used to generate separate score maps, which are titled MFEF1 and MFEF2. The only difference between them lies in that ConvLSTM is introduced into MFEF1, which is not utilized in MFEF2. Then, the two score maps are fused with different weights to generate the final score map, which is processed using the Softmax function for the extraction of water and shadow. The proposed framework has integrated CNN, DensNet, ResNet, ConvLSTM, and GCN to achieve enhanced performance in water and shadow extraction from SAR images. 

Compared with eight other frameworks in experiments, the presented MRDED framework achieves the best classification results for water and shadow, though there are still some very small misclassified areas. The PA for water, shadows, and background reaches 80.12%, 88%, and 95.16%, and their IoUs are 73.88%, 77.11%, and 90.49%, respectively. The overall accuracy for the three types of targets (water, shadows, and background) are 92.44% in PA, 87.86% in MPA, and 80.49% in MIoU. Therefore, MRDED presents satisfactory extraction performance for water and shadows from SAR images. Following this paper, researchers who are interested in the extraction and classification of SAR images can introduce other networks or modules to replace the existing MRDED components according to the various needs. For example, the GLGCM part could be substituted by other texture extraction algorithms, such as the Gabor transformation. The Decoder part can also be improved by integrating more modules with similar structure to MFEF1 and MFEF2. The performance of MRDED is very reliable even if SAR images were acquired from different sensing platforms (e.g., satellites, airplanes, and UAVs), at different resolutions, and with different geometric attributes. Because MRDED is based on the fusion of multiscale SAR features via advanced deep neural networks. To summarize, MRDED can be widely applied to perform classification of SAR images, in addition to water and shadow.

## Figures and Tables

**Table 1:** The network configuration of the Encoder part.

Layer Name	Output Size	101-Layer
Conv1	256 × 256	7×7, 64, stride 2
		3×3 max pool, stride 2
Conv2_x	128 × 128	$\left[\begin{array}{c} \begin{array}{c} 1\times 1,\;\;\;64\\ 3\times 3,\;\;\;64 \end{array}\\ 1\times 1,\;\;\;256 \end{array}\right]\times 3$
Conv3_x	64 × 64	$\left[\begin{array}{c} \begin{array}{c} 1\times 1,\;\;\;128\\ 3\times 3,\;\;\;128 \end{array}\\ 1\times 1,\;\;\;512 \end{array}\right]\times 4$
Conv4_x	32 × 32	$\left[\begin{array}{c} \begin{array}{c} 1\times 1,\;\;\;256\\ 3\times 3,\;\;\;256 \end{array}\\ 1\times 1,\;\;\;1024 \end{array}\right]\times 23$
Conv5_x	16 × 16	$\left[\begin{array}{c} \begin{array}{c} 1\times 1,\;\;\;512\\ 3\times 3,\;\;\;512 \end{array}\\ 1\times 1,\;\;\;2048 \end{array}\right]\times 3$

**Table 2:** The experiments for the adjustment of the number of feature maps.

Number of the Feature Maps	MIoU	Time (s)
256, 256, 256, 512	74.2	101
128, 128, 128, 256	74.2	70
64, 64, 64, 128	74.1	56
32, 32, 32, 64	73.8	52

**Table 3:** The classification accuracy of the first set of experiment.

Framework	PA	MPA	MIoU
**Encoder_MFEF2**	0.9101	0.8500	0.7651
**Encoder_MFEF1**	0.9145	0.8561	0.7699
**Lee_Encoder_MFEF1**	0.8885	0.7949	0.7208
**GLGCM_Encoder_MFEF1**	0.9104	0.8617	0.7759
**GLGCM_Encoder_MFEF1_2**	0.9206	0.8667	0.7918
**MRDED**	0.9244	0.8776	0.8049

**Table 4:** The classification accuracy of the different frameworks.

Framework	Water		Shadow		Background		PA	MPA	MIoU
	PA	IoU	PA	IoU	PA	IoU			
**RefineNet**	0.6276	0.6058	0.8829	0.7538	0.9536	0.8932	0.9098	0.8214	0.7509
**FCN-8s**	0.4011	0.3786	0.6764	0.6176	0.9814	0.8537	0.8695	0.6863	0.6166
**R-101_FCN**	0.5790	0.5576	0.6773	0.6389	0.9871	0.8557	0.8785	0.7478	0.6841
**L_K_M**	0.5548	0.5381	0.8083	0.7068	0.9719	0.8871	0.8981	0.7783	0.7107
**En_M1**	0.7147	0.6636	0.8955	0.7481	0.9399	0.8979	0.9145	0.8500	0.7699
**L_En_M**1	0.6968	0.6463	0.8646	0.6837	0.9082	0.8466	0.8803	0.8232	0.7255
**G_En_M1**	0.7748	0.6956	0.8684	0.7414	0.9419	0.8908	0.9104	0.8617	0.7759
**G_En_M1_2**	0.7573	0.7089	0.8995	0.7649	0.9433	0.9015	0.9206	0.8667	0.7918
**MRDED**	0.8012	0.7388	0.8800	0.7711	0.9516	09049	0.9244	0.8776	0.8049

## Appendix A

The specific experiments of MFEF1 & MFEF2 are described as follows:

The networks of MFEF1 and MFEF2 are trained and validated respectively, and then the features output from their last layer are weighted during testing. Finally, the features are processed to generate the final extracted results.

*Step 1: training stage.*

Perform separate training on MFEF1 and MFEF2 and select the models for testing based on the validated results.

*Step 2: testing stage.*

The image used for testing is cropped into small images using a sliding window of 512 × 512 and a step of 256.

**for** iteration = 1:num (images) do

$$y_{1}=F_{MFEF1}\left(\omega_{1},x\right)$$

$$y_{2}=F_{MFEF2}\left(\omega_{2},x\right)$$

where $\omega_{1}$, $\omega_{2}$ are the models of the networks of MFEF1 and MFEF2 respectively, and $y_{1}$ and $y_{2}$ are the score maps of MFEF1 and MFEF2 respectively. *x* and *F* are the input image and the network function respectively.

**end for**

*Step 3: Fusion stage.*

(1) Weighting and fusion of the obtained score maps:
  $$y_{fuse}=\omega_{a}*y_{1}+\omega_{b}*y_{2}$$
  where $\omega_{a}$ and $\omega_{b}$ are the weights, and they obey $\omega_{a}+\omega_{b}=1$.
(2) Fuse the obtained score maps of the small regions to generate the corresponding score map of the large-scale image.
(3) Input the obtained score map matrix of the large-scale image to the Softmax function to generate the Belief map:
  *b = Softmax(y_fuse_*)
(4) Each pixel of the belief map has a confidence probability belonging to each category, then the category corresponding to the maximum confidence probability is selected as the classified category. Finally, the classification results are fused with colormap to generate the final extracted results.
